# Women as Workers

**Published:** 1892-09-10

**Authors:** 


### Women as Workers.

### I.—WHERE WOMEN MUST FAIL.

In the world's history, woman has played three different parts, one after the other. Once she was the involuntary worker; later on, she became the involuntary idler; now, she is the voluntary worker—and such, it is to be hoped, she will for ever remain. Her enforced idleness was manifestly but a passing phase in the development of the civilised world —a shame-faced acknowledgment of the wrong that had been done her, when she, as the weaker vessel, was compelled to undertake such work as proved distasteful or over-burdensome to the masculine mind or body. Then, it would have been useless to discuss whether this or that task was suitable for a woman, or how she acquitted herself at her work. If she could not carry a heavy load in one journey, as a man could do—why, she might just take two or three journeys over it; if she could do her work no more than passably, was not that far better than that her lord and master should have to stop basking in the sunshine, and take such a "beastly grind" (doubtless there was an old-world equivalent for this) upon himself? But now *nous avons changé tout cela.* Woman is the recognised companion and helpmeet, not the slave, of man; and as no good man wishes to put upon her work for which she is not really fitted, and no sensible woman wishes to take it, it is worth while to consider how she is acquitting herself in her new sphere—where she is lacking, and how far she is able to supply that which she now lacks. It is always well to see clearly what we can and what we cannot do; for then what we cannot do may be abandoned once and for all, while what we can, but do not at present, do, may be diligently striven after.

In the first place, then, women should make up their minds that where really hard and continuous work is concerned, they must not expect—except, perhaps, in very rare instances—to compete with men. This is generally acknowledged where physical labour is in question: but many women are reluctant to admit it with regard to mental labour, seeming to think that it implies an insult to woman's intelligence. It implies nothing of the kind. A man in delicate health is not insulted by being told that he is not equal to the amount of labour, physical or mental, which a man in robust health can perform; and his is as nearly as possible a parallel case to that of women. Anyone who knows anything of the structure and delicate physical organisation of a woman must see that there is naturally far more strain upon her than upon a man, and that therefore she is less fitted to endure any extra strain that may be put upon her. A man in average health may go for many weeks together without a single symptom of discomfort; a woman in average health—average, that is, for a woman—is safe to suffer, in one way or another, during the same time—that is to say, there will be days, and very likely days not a few, when from headache, backache, or some other discomfort, she will feel "not up to the mark." Not up to the mark! What does this mean but that, in the long run, woman must needs fail in a competition with man; for, though for a time she may, through sheer pluck, make up the necessary tale of bricks without the straw, *i.e.*, the health and strength which more favoured ones are allowed, it can only be done at the cost of injury to herself, and in the long run must fail. It has been well said that to make a great statesman—a great statesman, at any rate, of the present day—two things are required, mental strength and physical strength, the latter, indeed, being to the full as important as the former. If Mr. Gladstone's physical strength had not been as phenomenal as his mental activity, he would have been dead long ago. There is but a certain amount of force in each man or woman; and when that force is exhausted, whether by a physical or a mental strain upon it, it is of no more use trying to draw upon it, than it is to draw cheques upon a bank where we have no longer any account. Therefore it is that men are guilty, if they only knew it, of suicidal folly, when, finding themselves utterly overdone in mind, they think to recruit themselves by violent physical exercise; and therefore it is that the average woman does serious harm both to herself and to her work, if she ignores the fact that the strain which she has to put upon herself to go on with her work in spite of physical suffering or *malaise*, really means so much force taken away from that work.

It is quite right, then, and no more than natural, that where men and women are engaged in the same trade, as, for instance, in tailoring, those parts of the work which require the most intellect should be given—as they are given—to men. They can concentrate all their force upon the work, while women are often trying to bear up under some physical discomfort, which, however bravely they may try to combat it, must tell upon them nevertheless.

Then there are "nerves" to be reckoned with; and only a woman knows what it really means to have one's nerves "on edge," or realises the exact nature of that mysterious desire to "fly out of one's skin." But this we do know, that nerve discomfort is one of the most trying of all discomforts; and when we add this to the other troubles which woman is heir to, it is really pathetic to think of the great army of workers—governesses, clerks, type-writers, and so on, who sally forth day after day, headachy, backachy, jarred in nerves, and with no opportunity of trying woman's universal and most efficacious panacea—"lying down."

Nevertheless, many women love work, and all would probably be the happier for it. The remedy for their troubles, then, is not that work should be taken from them, but that they, and the world in general, should understand their capabilities, and see—firstly, that they do not take work, or any amount of work, beyond their strength; and, secondly, that they so order themselves and their work, that it shall be done efficiently, and at the same time at as little cost to themselves as may be. After all, women are but new-comers into the field of labour; they have their credit to keep up, or rather to win, their strength to husband, and their tools to learn the use of. It does not in the least follow that because a woman fails once, she must needs fail always; indeed, it stands to reason that she must have time to adjust herself to her new sphere; and until she has had that time, we can only say, speaking generally, that her power of hard work is not equal to man's, leaving it to time and to herself to show exactly what she can and what she cannot do.

